# Adsorption of Different Ionic Types of Polyacrylamide on Montmorillonite Surface: Insight from QCM-D and Molecular Dynamic Simulation

**DOI:** 10.3390/molecules28114417

**Published:** 2023-05-29

**Authors:** Xiaomin Ma, Xiaosong Sun, Ming Chang, Qingxia Liu, Xianshu Dong, Yuping Fan, Ruxia Chen

**Affiliations:** 1 Department of Mineral Processing Engineering, Taiyuan University of Technology, Taiyuan 030024, China; ma_xiaomin@126.com (X.M.); sxs18734852978@163.com (X.S.); dongxianshu@tyut.edu.cn (X.D.); 19880628fyp@163.com (Y.F.); ruxiachen0828@163.com (R.C.); 2 State Key Laboratory of Mineral Processing, Beijing 100160, China; 3 College of New Materials and New Energies, Shenzhen Technology University, Shenzhen 518118, China

**Keywords:** polyacrylamide, montmorillonite, adsorption, QCM-D, molecular simulation

## Abstract

This study investigates the interaction between montmorillonite and polyacrylamide (PAM) with different ionic types using quartz crystal microbalance with dissipation monitoring (QCM-D) and molecular dynamics (MD) simulations. The goal was to understand the effect of ionicity and ionic type on polymer deposition on montmorillonite surfaces. The results of the QCM-D analysis showed that a decrease in pH led to an increase in the adsorption of montmorillonite on the alumina surface. The ranking of adsorption mass on alumina and pre-adsorbed montmorillonite alumina surfaces was found to be cationic polyacrylamide (CPAM) > polyacrylamide (NPAM) > anionic polyacrylamide (APAM). The study also found that CPAM had the strongest bridging effect on montmorillonite nanoparticles, followed by NPAM, while APAM had a negligible bridging effect. The MD simulations showed that ionicity had a significant influence on the adsorption of polyacrylamides. The cationic functional group N(CH_3_)^3+^ had the strongest attraction interaction with the montmorillonite surface, followed by the hydrogen bonding interaction of the amide functional group CONH_2_, and the anionic functional group COO- had a repulsive interaction. The results suggest that at high ionicity levels, CPAM can be adsorbed on the montmorillonite surface, while at low ionicity levels, APAM may still be adsorbed with a strong coordination trend.

## 1. Introduction

Understanding the interaction between water-soluble polymer flocculants and clay mineral particles is important in various engineering fields. Considering the area of mineral/coal, tailings/oil, and sand/management [1,2,3,4], the use of polymer flocculants to promote the sedimentation and subsequent dewatering of fine clay-rich particles is an extremely important procedure of the overall suite of the production process. Regarding the area of flotation [4], the interaction between polymers and clay can be used for selective flocculation flotation. Considering other fields, such as environmental pollution, the nanocomposites made by polymer and clay particles contribute to the removal of heavy metal ions and hazardous organic matter from water [5]. Regarding all the cases, the interaction of polymers and clays plays a crucial role in the efficiency of the operation.

As a kind of high-swelling clay mineral, montmorillonite (Mnt) can easily exfoliate to smaller particles and then increase the hydration degree and viscosity, leading to the higher stability of suspension than other clay minerals, so montmorillonite is widely encountered in engineering fields [6]. Polyacrylamide (PAM) is a linear water-soluble polymer with high molecular weight [7]. It has been widely used as a flocculant in industrial wastewater to aid solid–liquid separation [8,9]. The flocculation mechanism between PAM and solid particles, such as charge neutralization, bridging, and electrostatic adsorption is considered to have very good potential and effective strategies [10]. Generally, PAM is synthesized by copolymerization of acrylamide (AM) and repeating units of organic ionized functional groups, and they can be negatively or positively charged depending on the specific functional groups present [11]. Specifically, it can be divided into non-ionic polyacrylamide (NPAM), anionic polyacrylamide (APAM), and cationic polyacrylamide (CPAM). C_3_H_7_ON, C_3_H_6_O_2_, and C_5_H_9_O_2_N^+^ are the basic structural units of these PAM, resulting in different adsorption polarities [12,13]. The ionicity of PAM is quantitatively characterized as the percentage of the number of ionized functional groups in the total number of acrylamide chains [14]. The ionicity is one of the important indexes of PAM quality control, which determines the ionoelectric strength of water-soluble PAM [15]. The research on the interaction between montmorillonite and different ionic types of PAM has important practical guiding significance for industrial application.

Currently, some studies have been conducted to understand the interactions between montmorillonite and water-soluble PAM. Experimental methods such as zeta potential, sedimentation rate, adsorption capacity, AFM, and CFM were used to provide experimental information to help understand the adsorption behavior of PAM and montmorillonite flocculation [16,17,18]. Akimkhan [19], Heller and Keren [20] measured the adsorption of PAM on montmorillonite by dilute solution viscometry. They observed that the pattern of interaction of the polymer with the montmorillonite surface is determined by the properties of the polymer, the prevalence of acid-base sites, and the mineral crystal structure. CPAM appears to be preferred over other reagents for treating suspended solids of montmorillonite, and charge neutralization may occur between the cationically charged CPAM and the negatively charged montmorillonite particle surface [21]. Meanwhile, the interaction between PAM and montmorillonite is hydrogen bonding, charge neutralization, bridging and electrostatic adsorption, which is supported by most researchers [22]. However, the adsorption mechanism of different ionic types of PAM and montmorillonite is still not very clear.

The quartz crystal microbalance with dissipation monitoring (QCM-D) is a valuable and reliable tool used for its high accuracy and sensitivity in characterizing phenomena and interactions at liquid/solid interfaces [23]. The technique has also been used in probing the adsorption of polymer on minerals or mineral alternatives. Alagha et al. [24] used QCM-D to study the adsorption characteristics of PAM on the surfaces of silica sensors representing kaolinite tetrahedrons and alumina sensors representing alumina octahedra, in order to explore the adsorption properties of PAM on anisotropic basal planes of kaolinite. Xu et al. [25] used QCM-D to study the adsorption properties of a homemade agent Chi-g-PAM on a silica sensor and analyzed the adsorption conformation of the polymer on the sensor surface. Al-Hashmi et al. [26] investigated the adsorption of PAM and its derivatives on silica surfaces. The adsorbed amount and adsorption rate from the highest to lowest are nonionic PAM > partially hydrolyzed PAM > sulfonated PAM. However, most of the current studies on the adsorption of agents on silica alumina sensors are not well-represented on real mineral surfaces, and the details of the adsorption behavior such as the adsorption bridging behavior between mineral particles and PAM agents have not been studied. At the same time, most of the research work failed to consider the influence of the ionicity of PAM on the interaction of minerals.

In this study, the interaction between PAM of different ion types and montmorillonite nanocrystals was studied in situ by QCM-D to investigate the adsorption strength and bridging effects of different types of PAM on montmorillonite nanoparticles. Molecular dynamics (MD) simulations are more accurate in assisting the study of the mechanism of action of polymers on mineral surfaces in aqueous solutions. Recently, molecular simulation has been widely used to study the surface properties of minerals and the adsorption mechanism with organic molecules [27,28]. Therefore, in this study, anionic, cationic, non-PAM molecular models with different ionicity were constructed to conduct MD simulation of adsorption on the montmorillonite surface, and the effects of ionicity and ion type on the deposition of polymers on the surface of montmorillonite were studied. The insights provided by this study will help to explain macroscopic adsorption and flocculation experiments and provide useful information for the molecular design and development of a more effective flocculant.

## 2. Results and Discussion

### 2.1. QCM-D Results

#### 2.1.1. QCM-D Results of the Montmorillonite Adsorption on the Alumina Sensor

**Figure 1:**
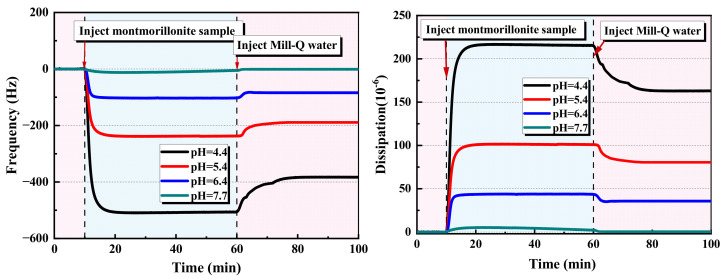
Adsorption of the montmorillonite on the alumina sensor at different pH.

The results of the adsorption kinetics of the montmorillonite on the alumina sensor at different pH are presented in Figure 1. The results show that there is a little shift in frequency and dissipation at pH = 7.7. This indicates that the montmorillonite particles are hardly adsorbed on the alumina sensor. At pH = 6.4, the decrease in the frequency by 102 Hz and the increase in dissipation by 44 × 10^−6^ indicate that a certain amount of montmorillonite particles are adsorbed on the alumina sensor. However, after the injection of water, there is an increase in frequency by 22 Hz and a decrease in dissipation by 9 × 10^−6^. This demonstrates the desorption of some particles from the sensor. When the pH decreases to 5.4 and 4.4, dramatic shifts in both the frequency and dissipation appear. Especially, regarding pH = 4.4, the shifts of frequency and dissipation are as large as −510 Hz and 220 × 10^−6^, respectively. This frequency shift indicates a strong adsorption of montmorillonite particles on the alumina surface. The relatively large increase in the dissipation shift indicates that considering the increase in the number of montmorillonite particles adsorbed on the sensor, the montmorillonite particles layer became softer and looser. This is because of the repulsion between the colloidal particles [29]. A clear trend of decreasing the pH value results in an increase in the frequency, dissipation, and amount of montmorillonite particle uptakes on the alumina surface.

**Figure 2:**
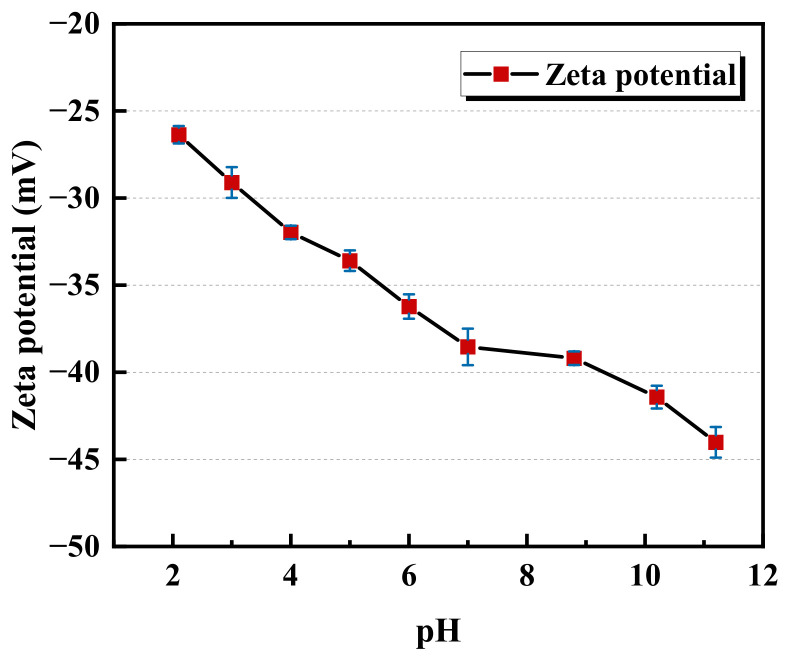
Zeta potential of the montmorillonite as a function of the pH.

The effect of the pH on the interaction between the montmorillonite and alumina surface can be explained by their zeta potential. Figure 2 shows the zeta potential of the montmorillonite as a function of the pH at the pH range 2–11. A monotonously decreasing trend of zeta potential with an increasing pH value is observed. Even at pH = 2.1, the montmorillonite particles have a large negative zeta potential (−26.5 mV). The isoelectric point of alumina reported in the literature varies widely but is generally located in the range of 6–9 [30]. Below the isoelectric point, the zeta potential of the alumina is positive, and considering the decrease in the pH, the zeta potential of the alumina increases. Therefore, at low pH, the attractive interaction between negatively charged montmorillonite particles and positively charged surfaces is enhanced.

#### 2.1.2. QCM-D Results of Polymers Adsorption on the Montmorillonite Pre-Adsorbed on the Alumina Sensor

**Figure 3:**
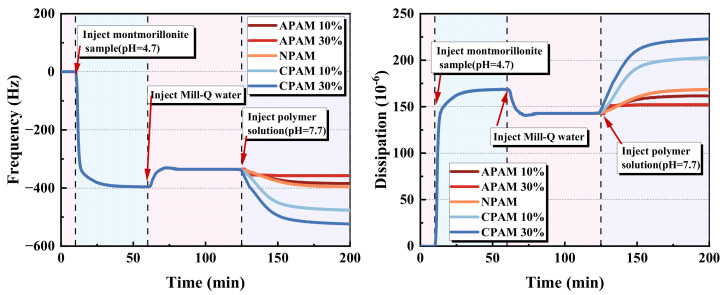
Adsorption of different polymer flocculants from 50 ppm solution on the montmorillonite pre-adsorbed on the alumina sensor.

**Figure 4:**
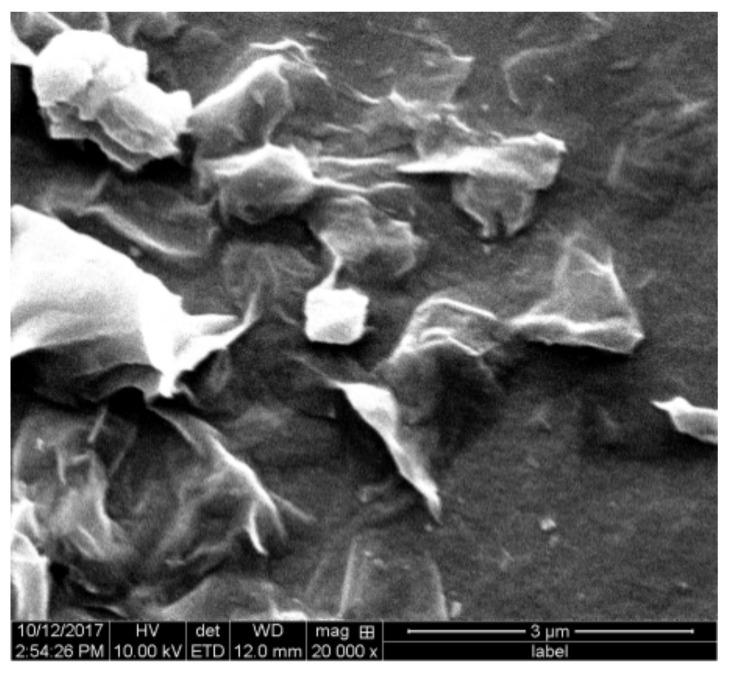
SEM of the montmorillonite (pH = 4.7) on the alumina sensor.

To study the adsorption of different ionic types of polyacrylamides on montmorillonite, the montmorillonite particles were first loaded onto an alumina surface at pH = 4.7. This pH value was selected because it could produce a large amount of montmorillonite adsorbed on the alumina sensor and prevent the sensor from being corroded by a strong acid. After the adsorption of the montmorillonite was stable, water was injected into the chamber to return the pH value for 1 h, and a polymer solution was introduced. Considering Figure 3, the montmorillonite particles cause a decrease in frequency by approximately 400 Hz. This indicates a large amount of adsorption of montmorillonite particles. After washing with water, the frequency increases by 70 Hz, implying that the weakly adsorbed particles are removed from the surface. Figure 4 is the SEM image of the alumina surface adsorbed with the montmorillonite particles. It can be observed that several montmorillonite particles cover the surface. Owing to the swelling properties of montmorillonite in water, its shape is irregular. Thereafter, a polymer flocculant solution is added. Figure 3 shows that the frequency and dissipation variation caused by APAM 30% is negligible, compared to those caused by APAM 10%, NPAM and CPAM. Moreover, APAM 10%, NPAM, CPAM 10% and CPAM 30% trigger frequency changes −21 Hz, −48 Hz, −61 Hz,−140 Hz and −190 Hz, and dispassion changes 19 × 10^−6^, 25 × 10^−6^, 62 × 10^−6^ and 79 × 10^−6^, respectively. These test results provide confirmation that the ionic degree and ionic type significantly influence the adsorption of PAM on montmorillonite surfaces.

**Figure 5:**
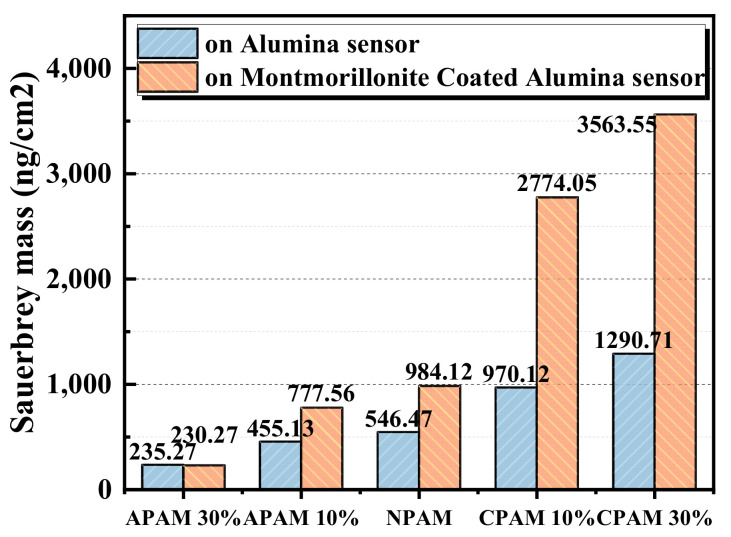
Adsorption mass of polymers flocculants on the alumina sensor and the montmorillonite pre-adsorbed on the alumina sensor.

We calculate and compare the adsorption mass of polymers on the alumina sensor and montmorillonite pre-adsorbed alumina sensor as shown in Figure 5. Figure 5 demonstrates that the adsorption mass of the CPAM 30% is the highest, and that of APAM is the lowest on both sensors. The adsorption mass of APAM 30% on the montmorillonite pre-adsorbed alumina sensor is a little lower than on the alumina sensor. The adsorption mass of NPAM on the alumina surface and montmorillonite pre-adsorbed alumina sensor is 0.54 mg/cm^2^ and 0.98 mg/cm^2^, respectively. The adsorption masses of the CPAM 30% on the alumina surface and alumina surface with montmorillonite adsorption layer are 1.29 mg/cm^2^ and 3.56 mg/cm^2^, respectively. The existence of montmorillonite can greatly enhance the adsorption of the CPAM and NPAM on the sensor.

#### 2.1.3. QCM-D Results of the Bridging Effect of Polymers on the Montmorillonite Particles

**Figure 6:**
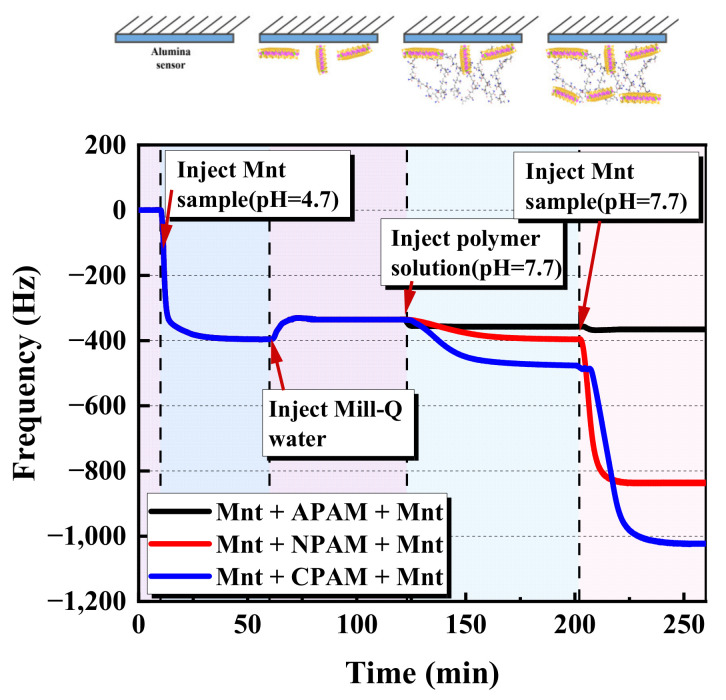
Bridging interaction of polymer flocculants on montmorillonite particles.

To further reveal the bridging interactions of polymer flocculants on montmorillonite particles, we selected APAM 30%, NPAM and CPAM 10% as representatives of different ionic types of PAM to conduct subsequent research with QCM-D as shown in Figure 6. After the polymer adsorption layer was formed (added for 70 min), the montmorillonite particles were injected into the QCM-D cell again. Considering the results of the CPAM cases, the shift of frequency is the largest (−580 Hz). This indicates the strong bridging effect the CPAM has on the montmorillonite particles. Moreover, NPAM exhibits a large shift in frequency and a strong interaction with the montmorillonite particles (−420 Hz). Regarding the APAM, its bridging interaction between the montmorillonite particles can be ignored based on the minor frequency change.

**Figure 7:**
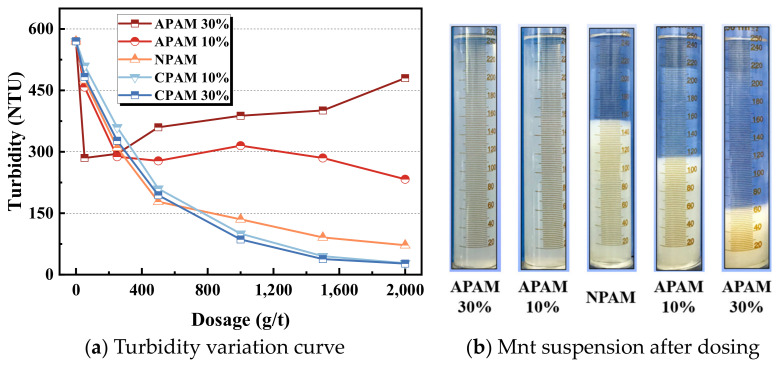
Clarification performance of polymers on montmorillonite suspension.

Figure 7 shows the clarification performance of the montmorillonite suspension as a function of the dosage and type of polymer. Figure 7b shows the turbidity of montmorillonite suspension with different reagents when the dosage is 1.5 kg/t. The turbidity of the supernatant is 580 after setting 20 min without adding any polymer. When the dosage is less than 300 g/t, the clarification effect of the APAM is better than that of NPAM and CPAM, and CPAM is the worst. This may be caused by the difference in the actual molecular weight. The higher the molecular weight, the smaller the number of polymers needed to produce the same clarification effect. With the dosage of APAM increasing, the turbidity increases, and the clarification effect decreases. The NPAM and CPAM usually require high dosages to achieve the clarification of montmorillonite. Regarding the increase in the dosage, the turbidity of NPAM and CPAM decreases, and the clarification effect increases. We found that when we added enough polymer, CPAM produced the best clarity, followed by NPAM, and APAM had the worst clarity. Generally, CPAM and NPAM reveal better flocculation and clarification effects than NPAM.

### 2.2. MD Simulation Results

#### 2.2.1. MD Simulation Results of NPAM Adsorption on Montmorillonite

**Figure 8:**
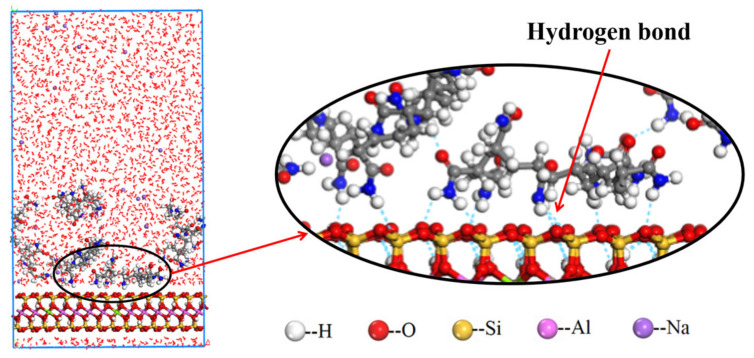
Equilibrium snapshot from MD simulation of the adsorption of NPAM on montmorillonite in the liquid phase at 298 K, 1 bar.

**Figure 9:**
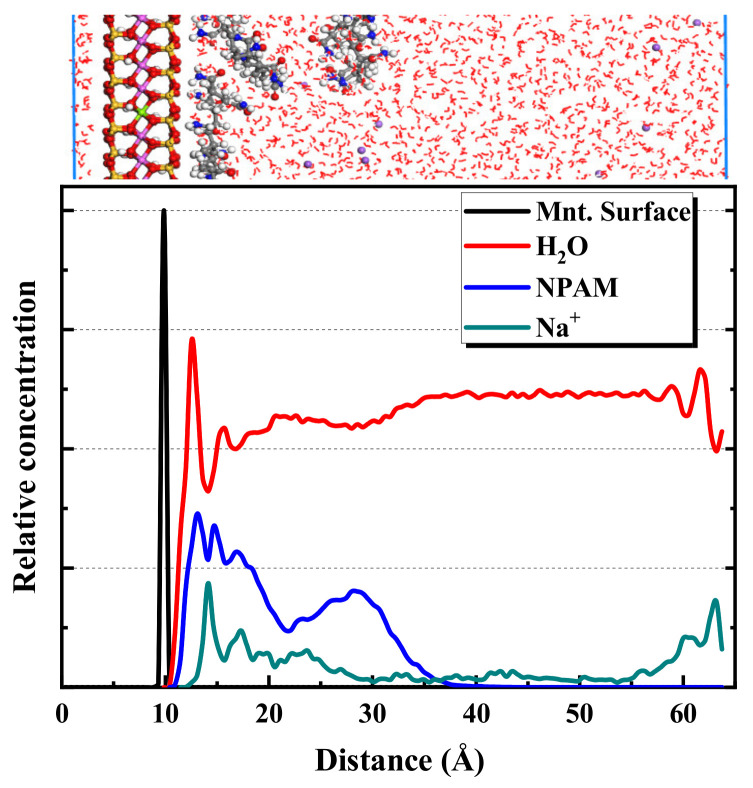
Distribution of the average relative concentration of components in montmorillonite/NPAM/water simulation system.

Figure 8 shows the equilibrium snapshot from the MD simulation of the adsorption of NPAM on montmorillonite in the liquid phase. The Figure 8 indicates that the hydrogen bonding interaction between the hydrogen atoms on the amide functional group of the NPAM molecule and the oxygen atom on the montmorillonite surface is critical for the adsorption of NPAM on the montmorillonite. The amido functional group marked by blue nitrogen atoms is approximately 2 angstroms closer to the montmorillonite surface than the C-C backbone marked by gray carbon atoms. The distance between the N atom and the horizontal plane where the oxygen atom is on the surface of the montmorillonite is about 2.7 Å. Intermolecular or intramolecular hydrogen bond is also widespread. This greatly affects the configurations of polymer molecules at the solid–liquid interface. Figure 9 shows the distribution of the average relative concentration of components in the montmorillonite/NPAM/water simulation system. The results show a distance of approximately 3 Å from the montmorillonite surface, and water forms a hydrated layer/shell. The NPAM forms an adsorption layer on the montmorillonite surface around the same position. The distance of Na^+^ from the montmorillonite surface is a little farther than that of water and NPAM. The distribution of components demonstrates that the interaction between the NPAM and montmorillonite surface is strong. Moreover, the hydrated shell cannot hinder the adsorption of NPAM.

#### 2.2.2. MD Simulation Results of APAM Adsorption on Montmorillonite

**Figure 10:**
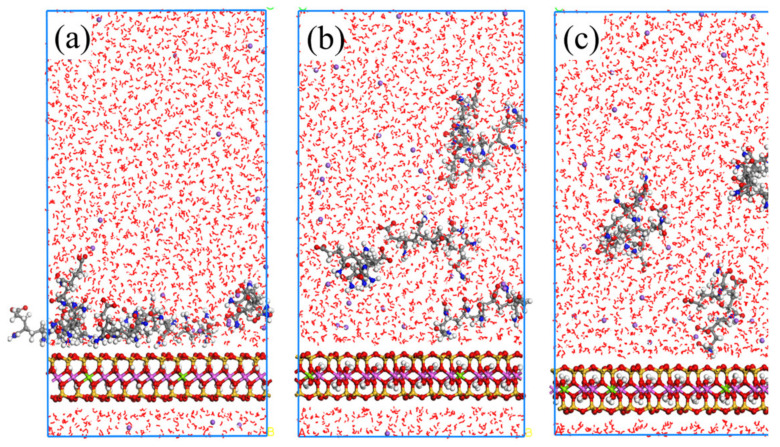
Equilibrium snapshot from MD simulation of APAM with ionicity of 12.5% (**a**), 25.0% (**b**), 37.5% (**c**) adsorption on montmorillonite from water at 298 K, 1 bar.

**Figure 11:**
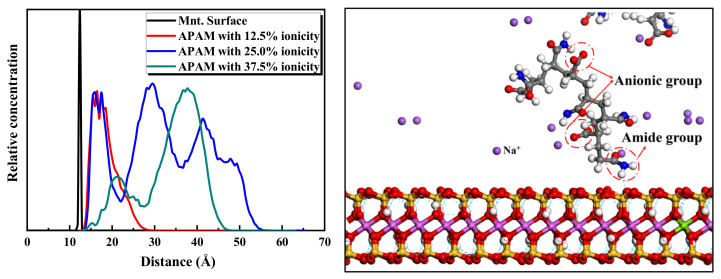
Relative concentration distributions of APAM with different ionicity in Montmorillonite/APAM solution system.

Considering Figure 10, regarding the case of ionicity 12.5%, the APAM molecules can still be adsorbed on the montmorillonite surface. However, when the ionicity of APAM increases to 25.0% and 37.5%, it tends to be freely dispersed in water rather than be adsorbed on the montmorillonite surface. Figure 11 shows the statistical distribution of different ionicity APAMs in the simulation system. The results clearly show that the increasing ionic degree of the APAM results in the weakening of its interaction with the montmorillonite. When the ionicity is 12.5%, APAM concentrates at position 3 Å from the surface. Considering ionicity 25.0%, APAM presents a multilayer distribution, indicating that some APAMs adsorb on the surface of montmorillonite, whereas some are dispersed in the water. When the ionicity is approximately 37.5%, APAM is mainly distributed 38 Å away from the montmorillonite surface. This indicates a very weak interaction and most APAMs cannot be adsorbed to the montmorillonite surface.

**Figure 12:**
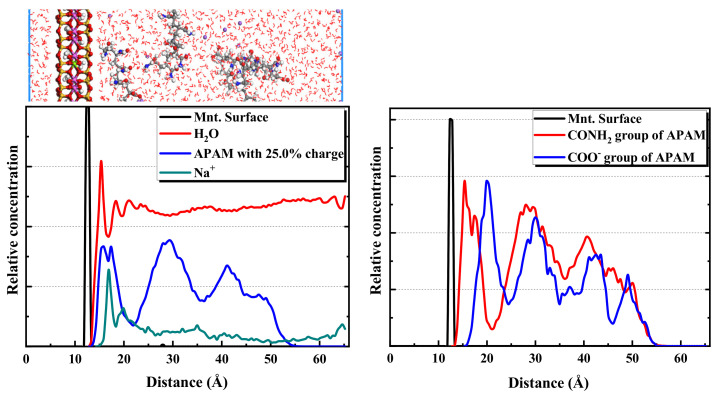
Relative concentration distributions of components and functional groups in montmorillonite/APAM (25.0% ionicity) solution system.

The 25.0% ionicity APAM simulation system is considered an example to reveal more details of the distribution of components and functional groups. The results are shown in Figure 12. Considering the figure, a hydration shell is formed at 3 Å from the montmorillonite surface, and the Na^+^ layer from the montmorillonite surface is a little farther than the water. The position of the APAM layer is quite close to that of the hydration shell. Although APAM presents a multilayer distribution, the amide groups CONH_2_ of the APAM molecules are closer to the montmorillonite surface than the anionic functional groups COO^-^. This indicates that the hydrogen bond between the amide group CONH_2_ and the montmorillonite surface promotes the adsorption of APAM on the montmorillonite surface. Nonetheless, the repulsion between the anionic group COO^-^ and the montmorillonite surface hinders the adsorption of APAM on the montmorillonite surface. When the content of the anionic group in APAM is low, the attraction between the amide group and the montmorillonite surface is dominant, and APAM can still adsorb on the montmorillonite surface. However, when the content of the anionic functional groups in APAM is high, the repulsion between the anionic groups and the montmorillonite surface is dominant. This makes it difficult for APAM to be adsorbed on the montmorillonite surface.

#### 2.2.3. MD Simulation Results of CPAM Adsorption on Montmorillonite

**Figure 13:**
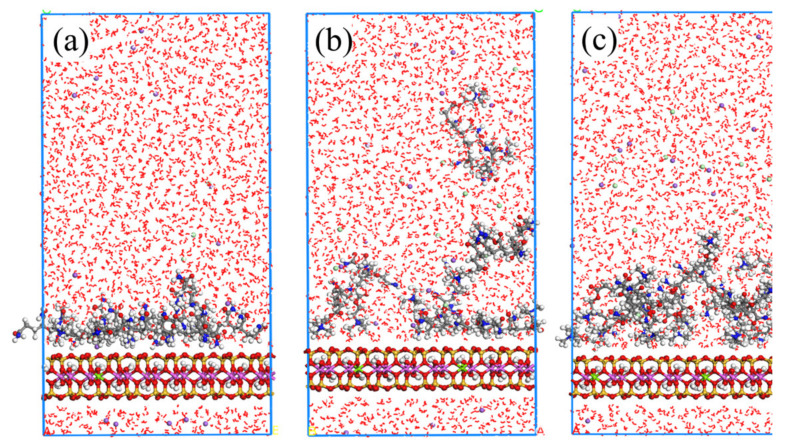
Equilibrium snapshot from the MD simulation of the CPAM with the ionicity of 12.5% (**a**), 25.0% (**b**), 37.5% (**c**) adsorption on the montmorillonite from water at 298 K, 1 bar.

**Figure 14:**
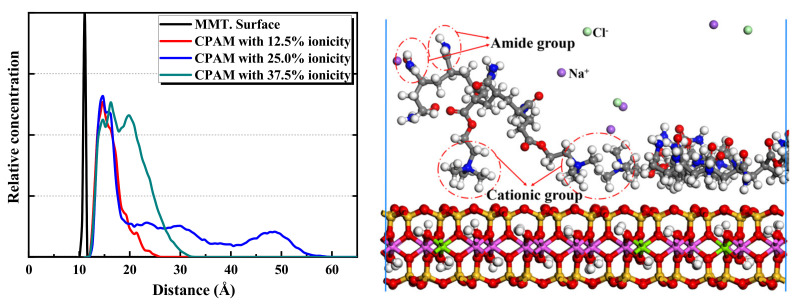
Relative concentration distributions of CPAM with different ionicity in the montmorillonite/CPAM solution system.

Figure 13 and Figure 14 present the simulation results of the CPAM system. Contrary to APAM, CPAM shows a good adsorption performance on the montmorillonite surface. However, the configuration of the adsorption layer changes with the variation of the polymer ionicity. Moreover, CPAM is concentrated at 3 Å from the surface of the montmorillonite. Regarding the 12.5% ionicity, a tight adsorption layer of the CPAM is formed. When the ionicity increases to 35%, the wide distribution peak of the CPAM indicates that the adsorption layer is relatively thick and loose. This is because of the increase in the number of ionicity functional groups. This leads to an increase in the intermolecular and intramolecular repulsion forces.

**Figure 15:**
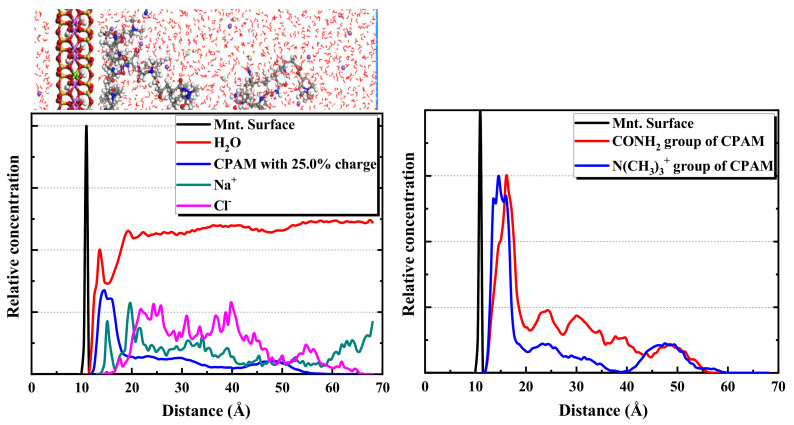
Relative concentration distributions of components and functional groups in montmorillonite/CPAM (25.0% ionicity) solution system.

Taking the CPAM system with 25.0% ionicity as an example, the distribution of components and functional groups was studied. As shown in Figure 15, water, CPAM and Na^+^ are closer to the surface of montmorillonite, whereas the Cl^-^ is far away from the surface. This is because of the negative ionicity of the Cl^-^ and the montmorillonite surface, resulting in a repulsion force. The N(CH_3_)_3_^+^ functional group in the CPAM is closer to the surface than the CONH_2_ functional group. The two main functional groups in CPAM can promote its adsorption with montmorillonite, and the adsorption interaction between the N(CH_3_)_3_^+^ and montmorillonite is stronger than that between the CONH_2_ and montmorillonite.

## 3. Materials and Methods

### 3.1. Materials 

Montmorillonite particles were obtained from Source Clays Repository (Sample SWy-2, Na-rich montmorillonite, The Clay Minerals Society, Purdue University). The polymers investigated were nonionic polyacrylamide (NPAM), anionic polyacrylamide (APAM) and cationic polyacrylamide (CPAM). This is because polyacrylamide-based polymers are commonly used in various water treatment industries [9,31,32,33,34]. Magnafloc 155 (APAM 10%), Magnafloc 5250 (APAM 30%), Magnafloc 351 (NPAM), Zetag 8110 (CPAM 10%) and Zetag 8140 (CPAM 30%) were purchased from Ciba (now BASF Corporation). The detailed characteristics of these commercial polymers are summarized in Table 1. Aluminum oxide (Al_2_O_3_) sensors were purchased from Biolin Scientific Inc. (US). Moreover, ACS-grade hydrochloric acid (HCl) and sodium hydroxide (NaOH) from Fisher Scientific (Hampton, NH, USA) were used to adjust the pH. Ultrapure Mill-Q water (18 MΩ cm^−1^ resistance) produced by the Millipore-UV Plus water purification system (Millipore Inc., Toronto, ON, Canada) is used throughout this study for all aqueous environments. 

**Figure 16:**
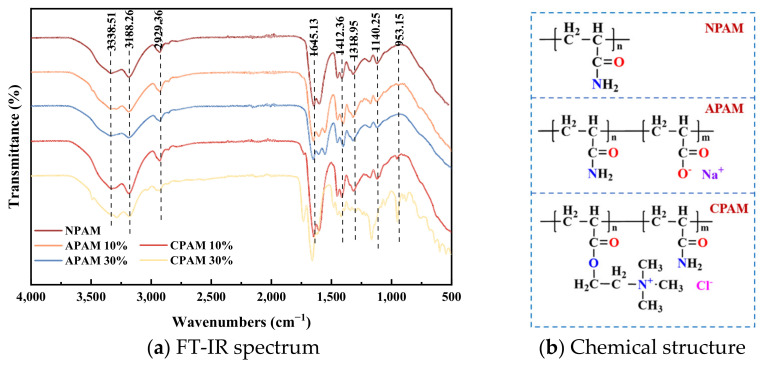
FT-IR spectrum and chemical structure comparison of anionic, cationic and nonionic polyacrylamides [36,37].

The FTIR spectra and chemical structure comparison of the anionic, cationic and nonionic PAM used in the experiment are shown in Figure 16. From the graphs, it can be observed that all PAM formulations exhibit an absorption peak at 3338 cm^−1^, corresponding to the N-H stretching vibration of the amide groups in the acrylamide structure. Due to the hydrophilic nature of the polymers, a stretching vibration peak of -OH appears at 3188 cm^−1^, possibly attributed to water molecules. The characteristic CH_2_ absorption peak connected to N is observed at 2923 cm^−1^. At 2132 cm^−1^, a non-symmetric stretching vibration peak of the methylene-CH_2_ group in the aliphatic tertiary amide structure is present. The stretching vibration peak of the C=O group in the amide structure unit can be seen at 1645 cm^−1^. Vibrational absorption peaks of the methylene-CH_2_ group are observed at 1474 cm^−1^ and 1274 cm^−1^. For the CPAM, an absorption peak of the quaternary ammonium group is observed at 953 cm^−1^. At 1140 cm^−1^, there is a methyl-CH_3_ peak connected to N^+^. As the degree of ionization increases, the corresponding structural peak becomes more pronounced. Regarding the APAM, two absorption peaks resembling carboxylic acid are observed around 1550 cm^−1^ and 1400 cm^−1^, representing the carboxylate-COONa structure.

### 3.2. Preparing the Montmorillonite Suspension

**Figure 17:**
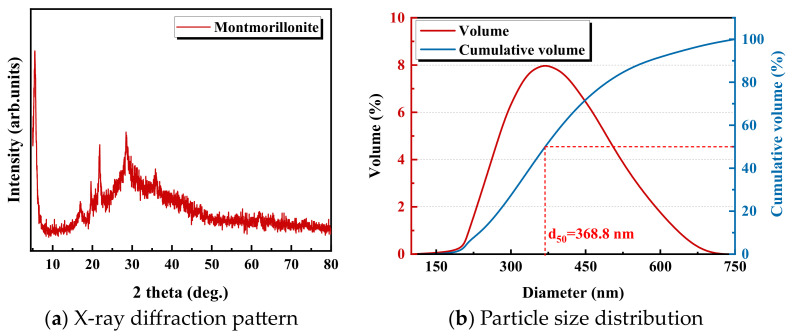
X-ray diffraction pattern and laser particle size distribution of the prepared montmorillonite particles.

The montmorillonite sample was first dispersed in Mill-Q water to prepare 8 wt% suspension and was stirred for three days to reach their swelling equilibrium. Thereafter, the suspension was treated with a sonic dismembrator (Model FB705, Fisher Scientific, Canada) at 30% of the maximum amplitude for 1 h. Finally, the montmorillonite suspension was left for another three days to enable the natural settling of the large particles. The remaining upper half suspension was stored and used in the QCM-D experiments. Figure 17a shows the XRD results of the prepared montmorillonite particles. The median diameter of the prepared montmorillonite suspension determined by NanoBrook ZetaPALS (Brookhaven Instruments Corporation, Holtsville, NY, USA) is 368.8 nm as shown in Figure 17b.

### 3.3. QCM-D Experiments

The experiments were conducted using QSense^®^ Analyzer (4-channel system) from Biolin Scientific (Gothenburg, Sweden). Moreover, QSensor QSX 309 Al_2_O_3_ (Aluminum oxide) was used in the experiments. Before each experiment, the alumina sensor was cleaned. First, the sensor with 99% ethanol was sonicated for 15 min. Subsequently, it was rinsed with Mill-Q water and carefully blow-dried with nitrogen gas. Next, the sensor was exposed to UV/ozone treatment for 10 min to remove hydrocarbon and organic contaminants. After the flow modules were installed, the four-sensor chamber was inverted to make the chip surface face down. The liquid was made to flow under the surface to ensure that the particles are absorbed, rather than being made to deposit by gravity onto the surface. The flow rate was 0.15 mL/min for all the experiments, and the temperature was set to 22 °C. Measurement data for frequency and dissipation were gathered simultaneously at several overtones (*n* = 1, 3, 5, 7, 9, 11 or 13). Owing to the stable and sensitive responses, the third overtones were used to present the results. [23,24,38,39]

#### 3.3.1. Adsorption Kinetic of the Montmorillonite on the Alumina Sensor

First, a starting baseline was established by the Mill-Q. Second, the prepared montmorillonite sample of the given pH was pumped into the cell of QCM-D. This operation lasts for 50 min to make sure the montmorillonite particles are fully absorbed on the sensor. Finally, Mill-Q water was pumped into the cell for 40 min to remove the weakly absorbed particles on the surface. During the testing, the pH of montmorillonite suspension is consistent with that of Mill-Q water.

#### 3.3.2. Adsorption Kinetic of Polymers on the Montmorillonite Pre-Adsorbed on the Alumina Sensor

**Figure 18:**
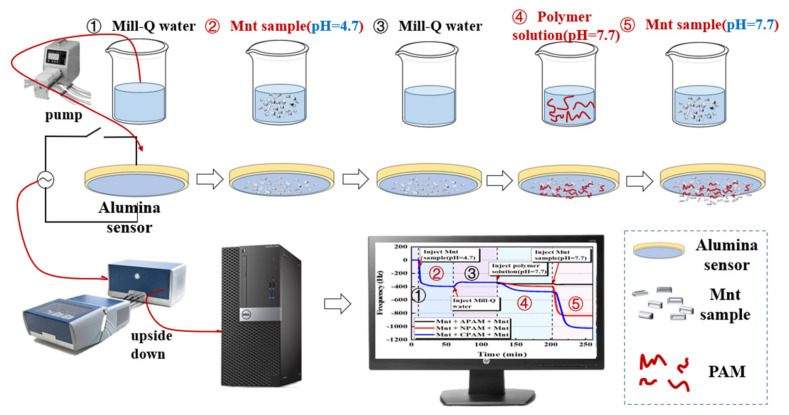
Diagram of the QCM-D test process.

First, a starting baseline was established by the Mill-Q. Then, the montmorillonite suspension of pH = 4.7 was pumped into the chamber of the QCM-D. This operation lasted for 50 min to make sure the montmorillonite particles were fully absorbed on the sensor. Next, the Mill-Q water was pumped into the cell for 1 h to remove the weakly absorbed particles on the surface and to adjust the pH close to neutral. Finally, the polymer solution (concentration 50 ppm and pH 7.7) was introduced. After the injection of the polymer for approximately 70 min, we injected the montmorillonite suspension (pH = 7.7) into the cell. Figure 18 shows a Diagram of the QCM-D test process.

### 3.4. Molecular Dynamic Simulation

**Figure 19:**
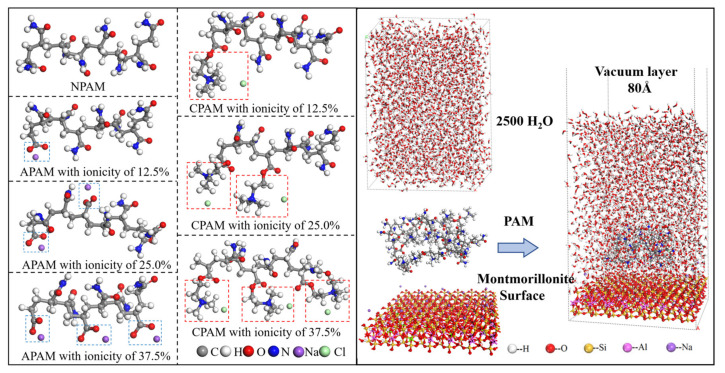
Construction of the PAM solution/montmorillonite surface molecules interface model.

The MD simulations were utilized to investigate the impact of ionicity and functional groups on the deposition of polymers onto the montmorillonite surface. In order to minimize the influence of extraneous factors, we adopted a simplified design for the simulation model. Figure 19 illustrates the structures of the anionic, cationic, and nonionic PAM with varying degrees of ionicity, as well as the montmorillonite model employed in the simulation. The polyacrylamide model contains eight monomers. The APAM and CPAM were built by substitutions of the anionic or cationic functional groups replacing the amido functional groups in the NPAM. When one of the eight amido functional groups is replaced by one anionic or cationic functional group, the ionicity of the APAM or CPAM obtained is 12.5%. When two or three of the eight amido functional groups are replaced, the ionicity of the APAM or CPAM obtained is 25.0% or 37.5%, Na ions were added to APAM and Cl ions to CPAM to compensate for the charge of the ionized PMA molecules. The montmorillonite model is consistent with the Na-rich montmorillonite sample used in the QCM-D experiments. It was obtained by isomorphous substitutions of Mg atoms replacing some of the Al atoms in the octahedral sheet. Moreover, Na^+^ was added to the layers to compensate for the ionicity. The general chemical formula for this dry montmorillonite model can be expressed as Na_0.75_[Mg_0.75_Al_3_](Si_4_O_10_)_7.5_(OH)_3.75_.

The MD simulation of the adsorption behaviors of the NPAM, APAM, or CPAM on the montmorillonite surface was performed within the Forcite module of Materials Studio 2019 (BIOVIA Dassault Systèmes, Paris, France). The size of the single lamella of montmorillonite was built at 3.6 × 3.6 nm^2^ in X and Y directions. Moreover, 2500 water molecules (density 1.0 g/cm^3^, thickness 57 nm) were placed above the montmorillonite surface to create a liquid environment. To obtain the system reaching equilibrium as quickly as possible, we initially placed eight polymer molecules randomly near the montmorillonite surface in each simulation. The vacuum layer thickness was set as 80 Å was added on the aqueous solution layer, in order to eliminate the interaction between the adjacent slabs. Therefore, the simulated system became a 36 × 36 × 147 Å3 supercell model are depicted in Figure 19, A total of 7 systems were simulated to study the adsorption of PAM on the montmorillonite/water interface, and the relevant information of PAM was collected. We performed geometry minimization and MD simulations under fine-quality settings in the software. Isobaric-isothermal ensemble (NPT) or canonical ensemble (NVT) was used for the equilibrium of different model systems. The short-range van der Waals interactions were truncated at 18.5 Å. Furthermore, the long-range electrostatics interactions were calculated using the Ewald summation method (accuracy of 1×10^−4^ kcal/mol). The time step was 1 fs. We controlled the system temperature and pressure by the Nosé–Hoover thermostat and Berendsen barostat, respectively. Generally, we ran the simulation at least 1000 ps NPT (at 298 K, 1 atm) to reach equilibrium and recorded the data in the other 500 ps NVT at the same pressure and temperature. All the calculations used the PCFF-Interface professional force field developed by [40]. This force field had been used in MD simulations of montmorillonite and had been proven to have high accuracy [28,41].

## 4. Conclusions

This study investigates the interaction between NPAM/APAM/CPAM and montmorillonite using QCM-D and MD simulations. The adsorption and bridging effect of polymers on montmorillonite nanoparticles are explored, and the effects of ionicity and functional groups of polymers are further analyzed. The main conclusions obtained from this study are summarized below.

(1) A decreasing pH results in an increase in the adsorption mass of montmorillonite on the alumina surface. This contributes to the enhancement of the electrostatic attraction between the positive alumina surface and the negative montmorillonite particles caused by the change in the zeta potential. Considering the alumina and pre-adsorbed montmorillonite particles’ alumina surface, the adsorption mass of polymers is ranked as CPAM > NPAM >APAM.
(2) Moreover, CPAM has the strongest bridging effect on montmorillonite nanoparticles, followed by NPAM, whereas APAM can hardly bridge the montmorillonite nanoparticles. The clarification test shows that when the dosage is high enough, the turbidity of CPAM is the lowest and the clarification effect is the best. This is consistent with the adsorption results from QCM-D.
(3) The ionicity density has a great influence on the adsorption of polyacrylamides and their derivatives. The cationic functional group N(CH_3_)_3_^+^ has the strongest attraction interaction with the montmorillonite surface, followed by the hydrogen bonding interaction of the amide functional group CONH_2_. The anionic functional group COO^-^ has a repulsive interaction.
(4) Furthermore, CPAM with different ionicity densities can be adsorbed on the montmorillonite surface through the N(CH_3_)_3_^+^ and CONH_2_ functional groups. It is more interracially active and tends to concentrate near the montmorillonite surface than the water layer. Regarding APAM, when the ionicity is low, CONH_2_ is dominant, and it can be adsorbed on the montmorillonite. Although the ionicity is high, the anionic functional group is dominant; thus, the adsorption is weakened.

## Figures and Tables

**Table 1:** Description of polymer flocculants provided by manufacturer and references.

Polymers	Ionic Type	Ionicity Density	Molecular Weight	Code Name
Magnafloc 155	Anionic	low (~10%)	High	APAM 10%
Magnafloc 5250	Anionic	Medium (~30%) [35]	High	APAM 30%
Magnafloc 351	Nonionic	Nano	High	NPAM
Zetag 8110	Cationic	Very low (~10%)	High	CPAM 10%
Zetag 8140	Cationic	Medium (~30%)	High	CPAM 30%

## Data Availability

Not applicable.
